# The genetic diversity of hepatitis A genotype I in Bulgaria

**DOI:** 10.1097/MD.0000000000009632

**Published:** 2018-01-19

**Authors:** Eleonora Cella, Elitsa N. Golkocheva-Markova, Diljana Trandeva-Bankova, Giulia Gregori, Roberto Bruni, Stefania Taffon, Michele Equestre, Angela Costantino, Silvia Spoto, Melissa Curtis, Anna Rita Ciccaglione, Massimo Ciccozzi, Silvia Angeletti

**Affiliations:** a Public Health and Infectious Diseases, Sapienza University, Rome, Italy; b NRL of Viral hepatitis, Virology department, National Center of Infectious and Parasitic Diseases (NCIPD), Sofia, Bulgaria; c Unit of Clinical Laboratory Science, University Campus Bio-Medico of Rome; d Viral Hepatitis Unit, Department of Infectious, Parasitic and Immune-Mediated Diseases; e Department of Cell Biology and Neurosciences, Istituto Superiore di Sanità; f Internal Medicine Department, University Hospital Campus Bio-Medico, Rome, Italy; g Department of Health, Human Performance and Recreation, Baylor University, Waco, TX, USA.

**Keywords:** Bulgaria, genotype I, HAV, hepatitis A virus, phylodynamics, phylogeny

## Abstract

*The purpose of this study was to* analyze sequences of hepatitis A virus (HAV) Ia and Ib genotypes from Bulgarian patients to investigate the molecular epidemiology of HAV genotype I during the years 2012 to 2014. Around 105 serum samples were collected by the Department of Virology of the National Center of Infectious and Parasitic Diseases in Bulgaria. The sequenced region encompassed the VP1/2A region of HAV genome. The sequences obtained from the samples were 103. For the phylogenetic analyses, 5 datasets were built to investigate the viral gene in/out flow among distinct HAV subpopulations in different geographic areas and to build a Bayesian dated tree, Bayesian phylogenetic and migration pattern analyses were performed. HAV Ib Bulgarian sequences mostly grouped into a single clade. This indicates that the Bulgarian epidemic is partially compartmentalized. It originated from a limited number of viruses and then spread through fecal-oral local transmission. HAV Ia Bulgarian sequences were intermixed with European sequences, suggesting that an Ia epidemic is not restricted to Bulgaria but can affect other European countries. The time-scaled phylogeny reconstruction showed the root of the tree dating in 2008 for genotype Ib and in 1999 for genotype Ia with a second epidemic entrance in 2003. The Bayesian skyline plot for genotype Ib showed a slow but continuous growth, sustained by fecal-oral route transmission. For genotype Ia, there was an exponential growth followed by a plateau, which suggests better infection control. Bidirectional viral flow for Ib genotype, involving different Bulgarian areas, was observed, whereas a unidirectional flow from Sofia to Ihtiman for genotype Ia was highlighted, suggesting the fecal-oral transmission route for Ia.

## Abstract 2

Supplemental Digital Content is available in the text

## 1 Introduction

*Hepatitis A virus* (HAV), a nonenveloped RNA virus belonging to the family Picornaviridae and genus Hepatovirus is the major cause of acute hepatitis throughout the world and contributes to substantial morbidity in both developed and developing countries.^[1]^ Recent estimates indicate a global incidence of 1.9% with 119 million cases of HAV infection.^[2]^

HAV is mainly transmitted by the fecal-oral route and can survive in water for a long time. Several epidemics have been observed as a consequence of contaminated drinking water and shellfish consumption.^[3–10]^ HAV real incidence is underestimated due to the under-reporting of this infection, which is characterized by asymptomatic and mild forms. Consequently, the epidemiological pattern can be evaluated by seroprevalence.

Based on nucleotide sequence analysis, human HAV is classified in 3 genotypes (I, II, and III), and subclassified in 6 subgenotypes (IA, IB, IIA, IIB, IIIA, IIIB). Genotypes and subtypes tend to show different geographic distribution.^[1]^

HAV epidemiology is highly age related: the majority of children aged <5 years are asymptomatic compared to older children and adults, who present with jaundice. As a consequence, the rate of patients requiring hospitalization increases with age, ranging from 21% in children <5 years to 53% among adults aged ≥60 years.^[11]^ In Western countries, the incidence of HAV infection has decreased among children as a result of improved sanitation and hygiene conditions. However, this decrease has generated an increasing proportion of adults who are susceptible to HAV^[12]^ and for whom clinical illness is more frequent and severe. This shift in the average age of infection has made HAV infection an increasingly important public-health problem in the Western world.^[2]^

The endemicity of HAV is ranked as high, intermediate, or low depending on the geographic areas. The level of endemicity can be defined as high in Africa, Asia, and Central, and South America, intermediate in Southern and Eastern Europe and the Middle East, and low in Northern and Western Europe, Japan, Australia, New Zealand, the USA, and Canada.^[2]^

In Europe, the epidemiology of HAV infection is highly variable with the majority of infections registered in Eastern European and Balkan region countries.^[13]^ Compared with the four-year average between 2009 and 2013, Hungary, Romania, and Slovakia reported large increases in the number of confirmed cases, while large decreases were reported by Bulgaria (http://www.euro.who.int).

Currently, according to the European Center for Disease Control and Prevention (ECDC), Bulgaria may be considered a country in transition phase from an intermediate to a low endemicity profile. However, the epidemiology of HAV in this country is uncertain due to the lack of quality studies. Molecular analysis of HAV isolates is needed to understand the demographic, social, and environmental factors that are giving rise to the current epidemiological patterns in Bulgaria.

In the present study, for the first time, sequences of HAV I genotype from Bulgarian patients were analyzed by Bayesian phylogenetic and migration patterns were analyzed to investigate the molecular epidemiology of HAV genotype I, circulating in Bulgaria during the years 2012 to 2014, and to evaluate the viral gene flow between different Bulgarian towns.

## 2 Methods

### 2.1 Patients and study design

One hundred and five serum samples were collected by the Department of Virology of the National Center of Infectious and Parasitic Diseases in Bulgaria. Samples were collected in several towns/villages throughout Bulgaria from patients with symptoms of acute hepatitis during 2012 to 2014. Among them, 91.4% were Bulgarian, and 51.4% were males. The mean age of the patients was 21 years, ranging from 2 to 78. All cases were locally diagnosed as HAV infections on the basis of specific IgM antibody detection. HAV RNA detection and sequencing were carried out in Italy at the National Reference Laboratory for Hepatitis Viruses—Istituto Superiore di Sanità (NRL-ISS). The study was approved by the Ethics Committee at the National Center of Infectious and Parasitic Diseases, Sofia, Bulgaria.

### 2.2 Nested RT-PCR and sequencing

Viral RNA was extracted from 140 μL serum by using the QIAmp viral RNA extraction kit (Qiagen, 129 Hilden, Germany). One-sixth of the extracted RNA (10 μL) was reverse transcribed by the SuperScript III First-Strand Synthesis System for RT-PCR (Invitrogen) with random hexamers. Nested PCR was carried out as previously reported.^[14]^

Double-strand sequencing of purified PCR products obtained from human sera was carried out by using the GenomeLab DTCS Quick Start Kit and an automated DNA sequencer (Beckman Coulter, Inc., Fullerton, CA). The sequenced region encompassed the VP1/2A region of HAV genome (460 nt, positions 2915 to 3374 in the HM-175 reference sequence Acc. No. NC_001489).^[15]^

### 2.3 Phylogenetic dataset and alignment

For the phylogenetic analyses 5 datasets were built. The first one included 103 HAV VP1-2A sequences from Bulgaria plus 13 reference sequences downloaded from NCBI (http://www.ncbi.nlm.nih.gov/genbank) for genotyping.

The second data set included 27 HAV VP1-2A sequences from Bulgaria, classified in the first dataset as Ib, plus 34 European HAV Ib VP1-2A sequences downloaded from NCBI (http://www.ncbi.nlm.nih.gov/genbank/). This dataset was used for Bayesian phylogenetic analysis. These sequences were downloaded from NCBI according to the following inclusion criterion: (1) known sampling country and (2) known sampling year.

The third dataset, including only the 27 HAV Ib VP1-2A Bulgarian sequences, was used to investigate the viral gene in/out flow among distinct HAV subpopulations in different geographic areas. Moreover, this data set was useful to build a Bayesian dated tree.

The fourth data set, including 27 HAV VP1-2A sequences from Bulgaria classified by first dataset as Ia and randomly chosen excluding clonal sequences plus 54 HAV Ia VP1-2A sequences, was used for Bayesian phylogenetic analysis. These sequences were downloaded from NCBI (http://www.ncbi.nlm.nih.gov/genbank/) following the same criteria of the second dataset.

The fifth dataset, including only the 27 HAV Ia VP1-2A Bulgarian sequences, was used to investigate the viral gene in/out flow among distinct HAV subpopulations in different geographic areas and to build a Bayesian dated tree.

All of the sequences were aligned using ClustalX software followed by manual editing using the Bioedit program v7.2.5, as already described.^[16]^

### 2.4 Likelihood mapping analysis

The phylogenetic signal of the datasets was investigated by means of likelihood mapping analysis of 10,000 random quartets using the TreePuzzle program, as already described.^[17]^ In this analysis, groups of 4 randomly chosen sequences (quartets) were evaluated using maximum likelihood (ML). For each quartet, the 3 possible unrooted trees were reconstructed under the selected substitution model. The likelihoods of each tree were then plotted on a triangular surface, so that fully resolved trees fall into the corners and the unresolved quartets in the centre of the triangle (indicating a star-like signal). When using this strategy, if more than 30% of the dots fall into the center of the triangle, the data are considered unreliable for the purpose of phylogenetic inference.

### 2.5 Phylogenetic analysis

For phylogenetic analysis, the evolutionary model for the 5 datasets was chosen as the best-fitting nucleotide substitution model in accordance with the results of the hierarchical likelihood ratio test (HLRT) implemented in MODELTEST software (version 3.7).^[18]^

On the first data set, the maximum likelihood (ML) phylogenetic tree was estimated using the best-fitting substitution model estimated by ModelTest^[18,19]^ (TN93 + G) with IQTREE.^[20]^ The statistical robustness and reliability of the branching order within the phylogenetic tree were confirmed with the bootstrap analysis and the fast likelihood-based sh-like probability.

For the second and fourth dataset, the Bayesian phylogenetic tree was reconstructed by means of MrBayes^[21]^ using the GTR + G model of nucleotide substitution according to Modeltest selection. The evolutionary rate was estimated on the second dataset by using a Bayesian Markov Chain Monte Carlo (MCMC) approach (Beast v. 1.8.2, http://beast.bio.ed.ac.uk) and implementing the evolutionary model selected by ModelTest.^[22,23]^ Independent MCMC runs were carried out, enforcing both a strict and relaxed clock, with an uncorrelated log normal rate distribution and one of the following coalescent priors: constant population size, exponential growth, nonparametric smooth skyride plot Gaussian Markov Random Field (GMRF), and nonparametric Bayesian skyline plot (BSP).^[22,24,25]^ Marginal likelihoods estimates for each demographic model were obtained using path sampling and stepping stone analyses.^[26–28]^ Uncertainty in the estimates was indicated by 95% highest posterior density (95% HPD) intervals, and the best-fitting model for each data set was determined by calculating the Bayes factors (BF).^[26,29]^ In practice, any 2 models can be compared to evaluate the strength of evidence against the null hypothesis (*H*_*0*_), defined as the one with the lower marginal likelihood: 2*ln*BF < 2 indicates no evidence against *H*_*0*_; 2–6: weak evidence; 6–10: strong evidence, and > 10: very strong evidence. Chains were conducted for at least 50 × 10^6^ generations, and sampled every 5000 steps for each molecular clock model. The convergence of the MCMC was assessed by calculating the ESS for each parameter. Only parameter estimates with ESS's of > 250 were accepted. Maximum clade credibility trees were obtained from the trees’ posterior distributions with the Tree-Annotator software v 1.8.2, included in the Beast package.^[22,23]^

The estimated evolutionary rate was used as prior to set a Bayesian dated tree on the third and fifth dataset by using the evolutionary model selected by Modeltest (GTR + G) and comparing the demographic model and the molecular clock, as reported above. Statistical support for specific monophyletic clades was assessed by calculating the posterior probability (pp).

On the third and fifth dataset, population dynamics was also analyzed by comparing the following demographic models: constant population size, exponential growth, nonparametric smooth skyride plot Gaussian Markov Random Field (GMRF), nonparametric Bayesian skyline plot (BSP), and implementing a relaxed molecular clock model under the conditions described above.

### 2.6 Viral gene flow analysis

The MacClade version 4 program (Sinauer Associates, Sunderland, MA) was used to investigate the viral gene in/out flow among distinct HAV subpopulations within different geographic areas corresponding to the towns of Bulgaria (Botevgrad, Dolna Banja, Elin Pelin, Gabrovo, Kostinbrod, Novachene, Pernik, Shumen, and Sofia) using the third dataset.^[30]^ A one-character data matrix is obtained from the original data set by assigning to each taxon in the tree a one-letter code indicating its city of origin. Then, the putative origin of each ancestral sequence (i.e., internal node) in the tree is inferred by finding the most parsimonious reconstruction (MPR) of the ancestral character. The final tree length, that is, the number of observed migrations in the genealogy, can easily be computed and compared to the tree-length distribution of 10,000 trees obtained by random joining–splitting.

Observed genealogies significantly shorter than random trees indicate the presence of subdivided populations with restricted gene flow.^[30]^ Specific migrations among different towns (character states) were traced with the State changes and stasis tool (MacClade software), which counts the number of changes in a tree for each pairwise character state. When multiple MPRs were present (as in our data set), the algorithm calculated the average migration count over all possible MPRs for each pair. The resulting pairwise migration matrix was then normalized, and a randomization test with 10,000 matrices obtained from 10,000 random trees (by random joining–splitting of the original tree) was performed to assess the statistical significance of the observed migration counts.

## 3 Results

The phylogenetic signal tested for each dataset produced good results as evidenced by the percentage of dots falling in the central area of the triangles being lower than 30%: 20.5%, 26.7%, 23.30%, 18,9%, and 24,5% for the first, second, third, fourth and fifth dataset, respectively (Supplementary Figure 1 panel a, b, c, d). Consequently, all datasets contained sufficient signal for phylogenetic analysis.

The genotype characterization was achieved by the ML tree showing several clusters corresponding to the different genotypes (Supplementary Figure 2). Seventy-six (73.8%) HAV Bulgarian sequences clustered inside Ia clade while the remaining sequences (27 sequences, 26.2%) clustered inside the Ib clade. No sequences belonged to IIa, IIb, IIIa, and IIIb genotypes.

**Figure 1:**
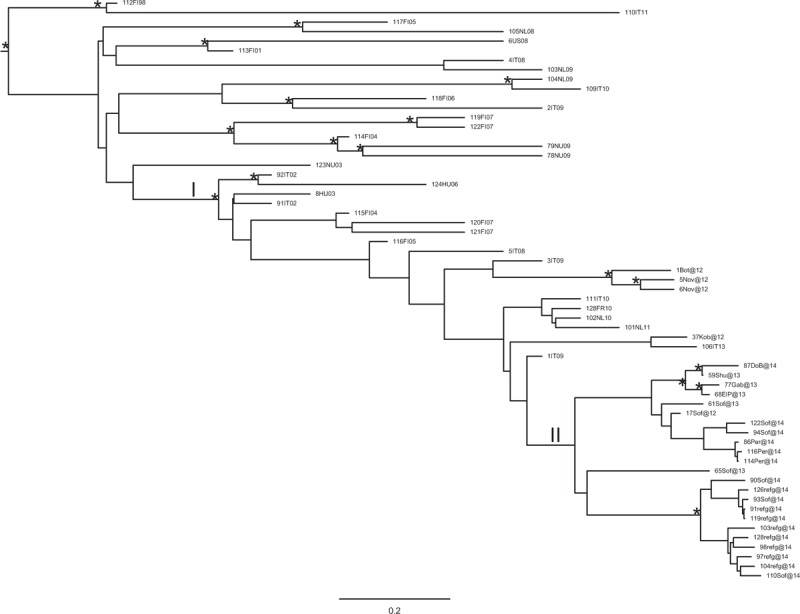
Bayesian phylogenetic tree of HAV Ib sequences of the second dataset obtained by MrBayes. One (∗) along a branch represents significant statistical support for the clade subtending that branch (posterior probability > 80%). The scale bar at the bottom indicates 0.2 nucleotide substitutions per site. HAV = hepatitis A virus.

The Bayesian phylogenetic tree constructed from the second dataset showed different clusters and clades statistically supported (pp ranging from 0.87 to 1) (Fig. 1). In the tree, all Bulgarian sequences were found in a statistically supported clade (I) including also other European sequences from Italy, Hungary, France, Netherlands, and Finland. Inside this clade 4 of the 27 Bulgarian sequences (14.8%), were intermixed with Italian sequences. Specifically, 3 Bulgarian sequences (1Bot@12, 5Nov@12, and 6Nov@12) formed a cluster with a South Italian sequence (3IT09); similarly, a Bulgarian sequence (37Kob@12) was found in cluster with a sequence from the Central Italy (106IT13).

The remaining 23 Bulgarian sequences (23/27, 85.2%), which represented the majority of the Bulgarian strains mostly from Sofia, formed a homogenous clade (II). Inside clade II, 2 supported clusters can be highlighted, 1 involving 4 sequences and the second including 11 sequences.

**Figure 2:**
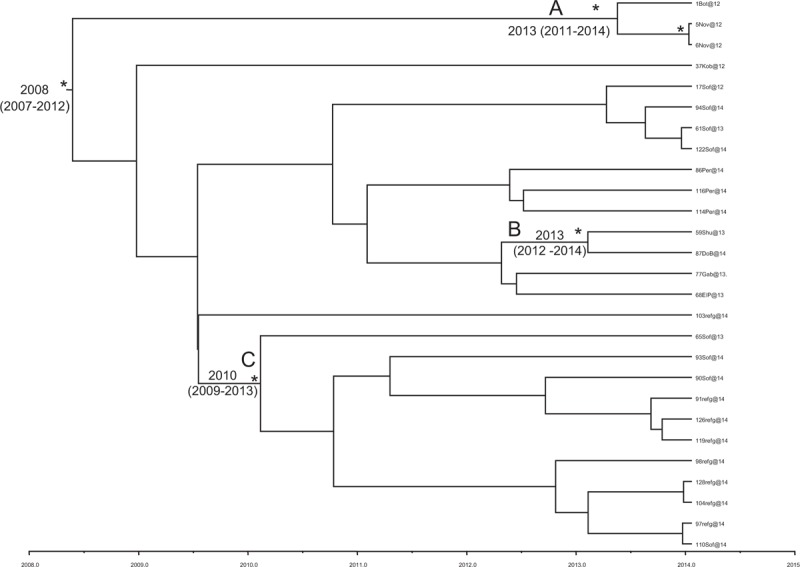
Bayesian time-scaled tree of Bulgarian Ib HAV sequences (third dataset). The time of the most recent common ancestor, with the credibility interval based on 95% highest posterior density interval (95% HPD), was reported in years. Scaled years are reported at the bottom of the figure. Two clusters (A, B) and one clade (C) are highlighted. HAV = hepatitis A virus, HPD = highest posterior density.

In Figure 2, the Bayesian phylogenetic tree in a calendar timescale performed on the third dataset has been represented. The Bayesian skyline plot demographic model with a relaxed molecular clock was selected as the most appropriate to describe the evolutionary history of HAV Ib Bulgarian sequences. The evolutionary rate used, estimated on the second dataset, was 1.85 × 10^−3^ substitutions site per year (95% HPD 1.32×10^−3^ − 2.4×10^−3^).

The root of the tree had a time of the most common recent ancestor (tMRCA) corresponding to 2008 (HPD 95% 2007–2012). Two clusters (A, B) and 1 clade (C) were highlighted. Cluster A included 3 sequences sampled in 2012: 1 from Botevgrad and 2 from Novachene This cluster dated back to 2013 (HPD 95% 2011–2014).

Cluster B included 2 sequences: 1 from Shumen, sampled in 2013, and 1from Dolna Banja in 2014. This cluster dated back to 2013 (HPD 95% 2012–2014). Clade C included 11 sequences: 4 sequences from Sofia, sampled in 2013 and 2014, and 7 sequences from symptomatic refugee patients hospitalized in Sofia in 2014. This clade dated back to 2010 (HPD 95% 2009–2013).

**Figure 3:**
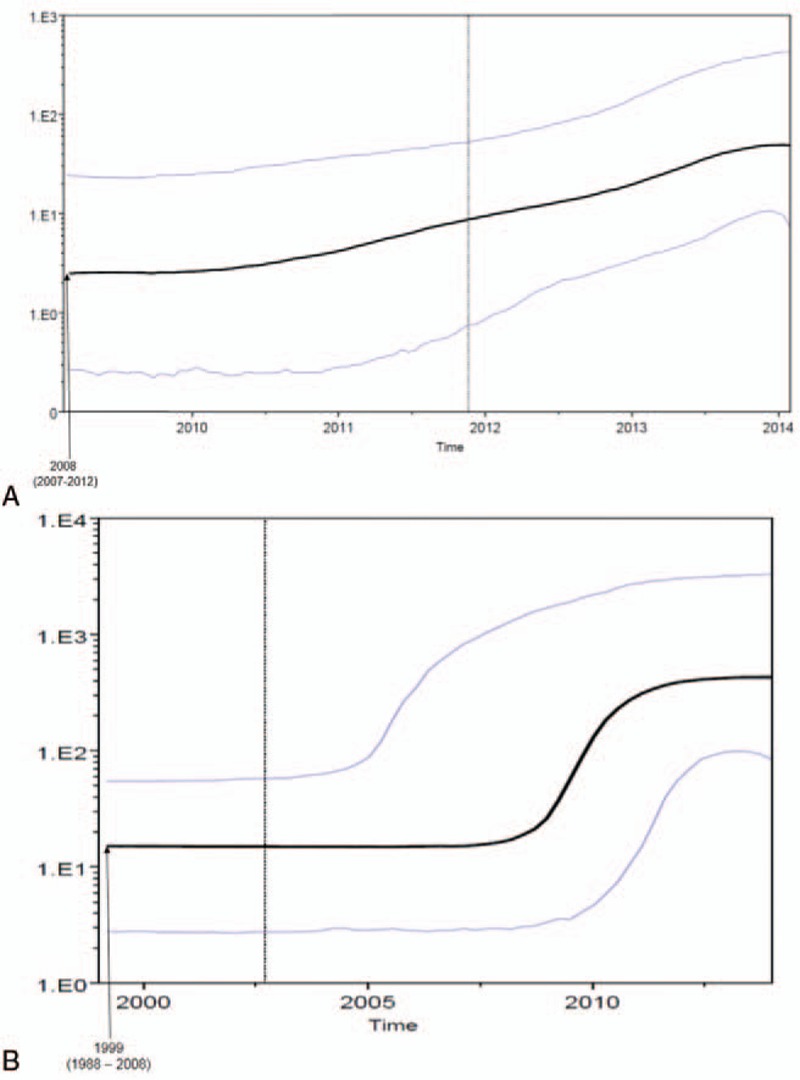
Demographic history of Bulgarian HAV genotype I (subtype Ib: panel a; subtype Ia: panel b). The demographic history was inferred by a nonparametric Bayesian skyline plot (BSP). The *y*-axis reports virus effective population size (Ne), a measure of genetic diversity representing the number of genomes effectively contributing to new infections, while the *x*-axis is time in calendar years. Black lines are median estimates; purple lines are 95% highest posterior density intervals. BSP = Bayesian skyline plot, HAV = hepatitis A virus.

The Bayesian skyline plot for the effective population size of HAV Ib and Ia Bulgarian sequences built by the third and fifth dataset is shown in Figure 3. A slow constant growth for Ib infection from 2010 to 2014 (Panel a) and an exponential growth for Ia infection number from 2009 until 2011 and the reaching of the plateau (panel b) are evident.

**Figure 4:**
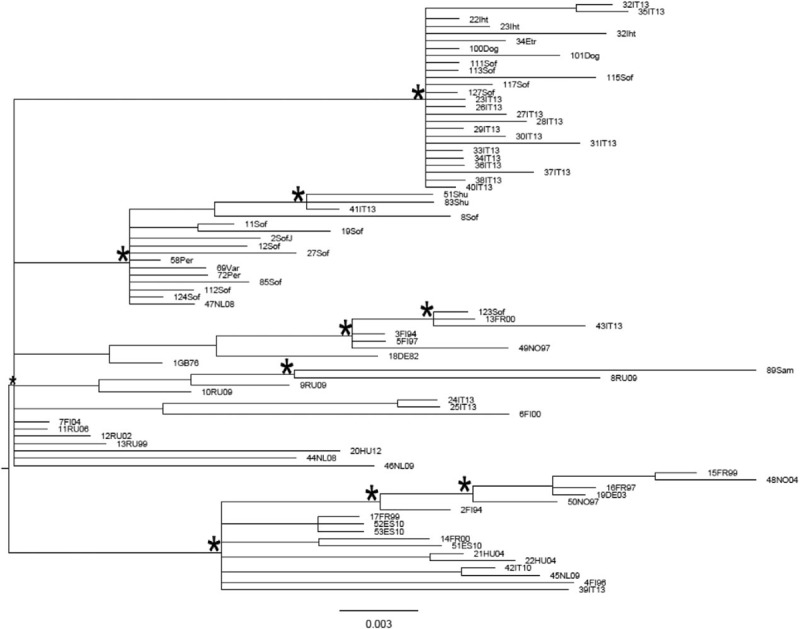
Bayesian phylogenetic tree of HAV Ia sequences of the fourth dataset obtained by MrBayes. One (∗) along a branch represents significant statistical support for the clade subtending that branch (posterior probability > 80%). The scale bar at the bottom indicates 0.003 nucleotide substitutions per site. HAV = hepatitis A virus.

In the Bayesian phylogenetic tree constructed on the fourth dataset for genotype Ia (Fig. 4) different statistically supported clusters and clades (posterior probability > 80%) are evident. All Bulgarian sequences were intermingled in statistically supported clades, including also other European sequences from different countries.

**Figure 5:**
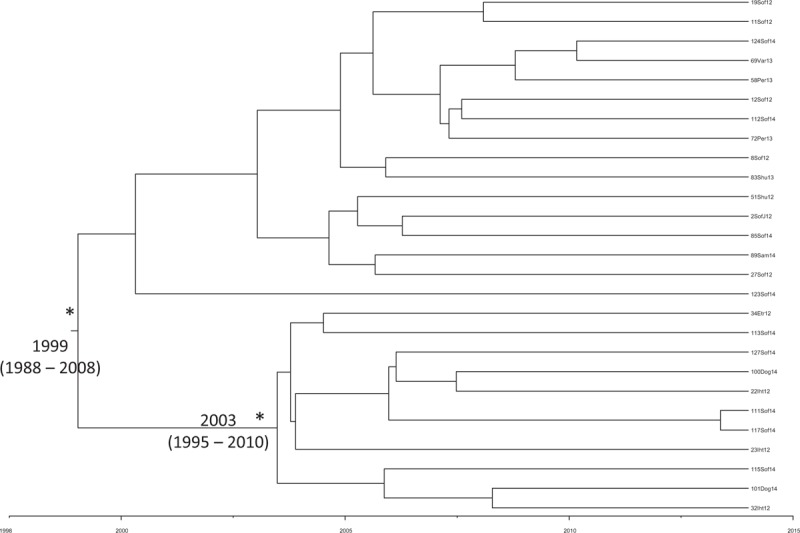
Bayesian time-scaled tree of Bulgarian Ia HAV sequences (fifth dataset). The time of the most recent common ancestor, with the credibility interval based on 95% highest posterior density interval (95% HPD), was reported in years. Scaled years are reported at the bottom of the figure. One (∗) along a branch represents significant statistical support for the clade subtending that branch (posterior probability > 98%). HAV = hepatitis A virus, HPD = highest posterior density.

The Bayesian phylogenetic tree in a calendar timescale performed on the fifth dataset is shown in Figure 5. The Bayesian skyline plot demographic model with a relaxed molecular clock was selected as the most appropriate to describe the evolutionary history of HAV Ia Bulgarian sequences. The evolutionary rate used, estimated on the fourth dataset, was 1.13 × 10^−3^ substitutions site per year (95% HPD 6.2 × 10^−4^ – 1.69 × 10^−3^). The root of the tree had a time of the most common recent ancestor (tMRCA) corresponding to 1999 (HPD 95% 1988–2008). One statistically supported cluster, dated back to 2003 (HPD 95% 1995–2010), was highlighted.

**Figure 6:**
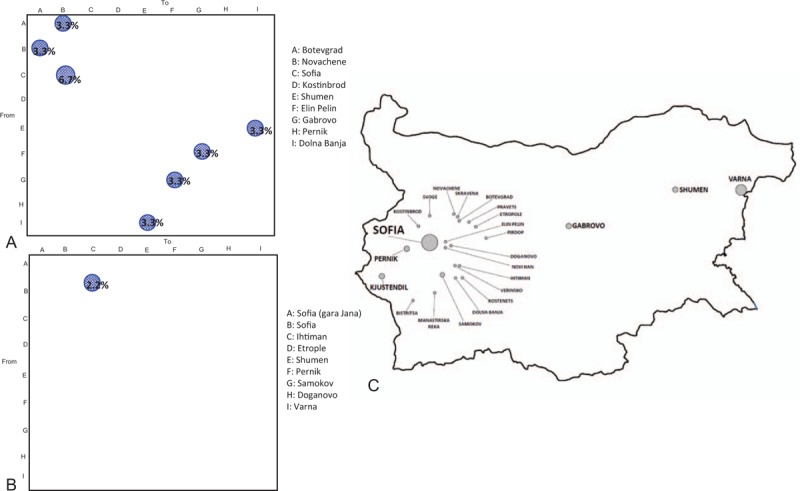
Migration pattern of HAV genotype I circulation in Bulgaria. The bubblegram shows the frequency of gene flow (migrations) in Bulgaria to/from different geographic areas (towns). The surface of each circle is proportional to the percentage of observed migrations. Migrations were inferred with a modified version of the Slatkin and Maddison algorithm. (Panel A) HAV subtype Ib (third dataset). A: Botevgrad; B: Novachene; C: Sofia; D: Kostinbrod; E: Shumen; F: Elin Pelin; G: Gabrovo; H: Pernik; I: Dolna Banja. (Panel B**)** subtype Ia (fifth dataset). A: Sofia (Gara Jana); B: Sofia; C: Ihtiman; D: Etrople; E: Shumen; F: Pernik; G: Samokov; H: Doganovo; I: Varna. (Panel C**)** map of Bulgaria highlighting the different area enrolled in migration patterns of HAV genotype I. HAV = hepatitis A virus.

The gene flow (migration) of HAV genotype Ib (third dataset) and Ia (fifth dataset) among different towns in Bulgaria is reported in Figure 6. For genotype Ib (panel A), the 9 towns considered were the following: A, Botevgrad; B, Novachene; C, Sofia; D, Kostinbrod; E, Shumen; F, Elin Pelin; G, Gabrovo; H, Pernik; I, Dolna Banja). For genotype Ia (panel B), the 9 towns considered were the following: A, Sofia (Gara Jana); B, Sofia; C, Ihtiman; D, Etrople; E, Shumen; F, Pernik; G, Samokov; H, Doganovo; I, Varna). The gene flow was investigated with a modified version of the Slatkin and Maddison method.^[30]^ The town of origin of each ancestral node (i.e., ancestral sequence) was inferred, using the maximum parsimony algorithm. The null hypothesis of panmixia (i.e., no population subdivision or complete intermixing of sequences from different geographical areas) was tested using a bubblegram. The migration flow among the different nine towns was then estimated as observed migration in the genealogy (Fig. 6). The null hypothesis of panmixia was rejected by the randomization test (*P* < .0001) for all the towns in the bubblegram.^[30]^

As shown in the bubblegram in Figure 6 panel A, the viral gene flow for genotype Ib indicated bidirectional exchanges involving 2 towns (Botevgrad and Novachene, 3.3% each). Similarly, this was observed in other towns: Shumen—Dolna Banja and Elin Pelin—Gabrovo (3.3%).

A unique flow from Sofia is directed to Novachene (6.7%). No viral gene flow from Kostinbrod and Pernik to the other towns was observed.

As shown in the bubblegram in Figure 6 panel B, the viral gene flow for genotype Ia indicated a unique flow from Sofia to Ihtiman (2.2%).

## 4 Discussion

Hepatitis A infection represents a serious public health problem in Bulgaria. Bulgaria is a country characterized by an emerging market economy. It is an important exporter of agricultural and food products, whereas it imports only industrial and chemical products. The country's main export partners are its neighbors and other European countries such as Germany, Italy, Romania, Belgium, Turkey, and Greece.

After the large HAV epidemic of 1991 in Bulgaria, the number of cases decreased until the years 2010 to 2012 when a serial epidemic peak of acute infection was observed affecting predominantly young people aged up to 19.^[31]^

Data from the ECDC surveillance^[13]^ defined Bulgaria as the European country with the highest number of hepatitis A cases registered during 2011 and 2012 with HAV outbreaks still occurring.

Currently, Bulgaria may be considered in a transition phase from an intermediate to a low endemicity profile; however, some uncertainties must be considered due to the likelihood of intranational variation of HAV seroprevalence.^[32]^

In 2009, Vatev et al^[33]^ reported a mean anti-HAV seroprevalence of 68.33% in 1 Bulgarian region (Plodiv).

These data were compared with 2 other Bulgarian regions, Sofia and Pleven (prevalence of anti-HAV IgG of 44.06% and 54.5%, respectively)^[32]^ where there were decreasing trends for HAV seroprevalence.

In the present study, phylogenetic analysis of Bulgarian HAV Ia and Ib strains circulating in 2012 to 2014 was reported to give deep insight into the epidemiological picture of HAV infections in this country.

Phylogenetic analysis of HAV Ib Bulgarian sequences identified several clusters, revealing only in few cases intermixing between Bulgarian and European sequences. The intermixing could probably be due to an exchange of contaminated food and water among Bulgaria and other European countries.

Although, a clade (II) including only Bulgarian sequences was also detected. This could indicate that the Bulgarian epidemic is partially compartmentalized and possibly originated from the introduction of a limited number of viruses, followed by spread through fecal-oral local transmission.

The time-scaled phylogeny reconstruction showed that the root of the tree originated in 2008, suggesting that this HAV Ib epidemic entered Bulgaria in that period. Moreover, 3 different introductions (cluster A, B, and C) into Bulgaria at different times were found. Specifically, cluster A originated in 2013 and included 3 sequences, sampled in Botevgrad and Novachene. Botevgrad and Novachene, a city and a village, respectively, both located in the Sofia region, are very near to each other. Therefore, it is possible to hypothesize an exchange between these 2 locations.

Cluster B originated in 2013 and included 2 sequences, 1 from Shumen and 1 from Dolna Banja, 2 cities located in different regions of Bulgaria (Shumen and Sofia region, respectively). Clade C, dating back to 2010, included 11 sequences from Sofia, 4 of which were sampled in 2013 to 2014 and 7 from symptomatic refugee patients hospitalized in Sofia in 2014. Since these refugee patients were hospitalized with clinical symptoms, it is possible to hypothesize that these patients were infected during their stay in Sofia.

Phylogenetic analysis of HAV Ia Bulgarian sequences identified several clusters where the Bulgarian sequences were intermixed with the European ones. This could suggest that Ia epidemic is not restricted to Bulgaria but that this HAV genotype circulates across different European countries.

The time-scaled phylogeny reconstruction showed that the root of the tree originated in 1999 with a second epidemic entrance in 2003. This evidence could suggest that Bulgarian HAV Ia strains began circulating in the country before the genotype Ib.

The Bayesian skyline plot for the effective population size of HAV Ia and Ib in Bulgaria showed a difference between the 2 genotypes. The number of infections caused by genotype Ib showed a slow but continuous growth over time, probably due to the maintenance of the oral-fecal circuit within the country. For the genotype Ia, the number of infections after an exponential growth reached a plateau, suggesting better control, probably because this infection could be a consequence of contaminated food.

In the gene flow analysis is noteworthy the bidirectional flows for HAV Ib genotype, involving Botevgrad and Novachene. Novachene is located in South-Western region of Bulgaria and is part of Botevgrad municipality. These 2 locations were geographically linked and located a few kilometers away from each other. This could explain the gene flow analysis reported above. A 3.3% of bidirectional gene flow was also observed between Shumen and Dolna Banja and 6.7% from Sofia to Novachene. Other towns were involved in the gene flow: Shumen—Dolna Banja and Elin Pelin—Gabrovo (3.3%) indicating the central role of these locations in the dispersion of virus flow. Interestingly, for HAV genotype Ia a 2.2% unidirectional virus flow has been observed from Sofia to Ihtiman. Ihtiman is a town in Western Bulgaria, and it is part of Sofia province. It is located in a valley at a distance of 48 km from Sofia, close to the Trakiya motorway connecting Sofia, Plodiv, and Burgas on the Black Sea Coast. This unidirectional viral flow from Sofia to Ihtiman could suggest that the transmission of HAV genotype Ia could not be due to the oral-fecal circuit.

Strengths of the present study are represented by the use of sophisticated molecular epidemiological tools to estimate the origin and evolution of HAV epidemic in Bulgaria, whereas a limitation could be the lack of retrospective data collection by classical epidemiological investigation.

In conclusion, multiple factors are likely to be involved in determining the different epidemiological picture of HAV genotype I infection in Bulgaria, such as population movement, food trade, and exchange of contaminated food. Interactions between these factors may have contributed to the spread of the virus.

In conclusion, this study focusing on the epidemiological history and migration pattern of HAV genotype I in Bulgaria was crucial to understand the dynamics of virus circulation and to implement preventive measures useful for public health.

## 5 Author contributions

CM and AS conceived the project hypothesis with contributions from G-ME, T-BD, CAR, CE, and GG conceived and performed the evolutionary analyses with contributions by CM and AS. CE, CM, and AS wrote the paper with contributions from BR, TS, EM, CA, SS, CAR, and CM. All authors agreed on interpretation of results, and revised the manuscripts.

## Acknowledgment

In memory of our friend Pavel Teoharov.

## Supplementary Material

Supplemental Digital Content
