# Supplementary material for: The genetic diversity of hepatitis A genotype I in Bulgaria
Source: Medicine (Baltimore). 2018 Jan 19;97(3):e9632. doi: 10.1097/MD.0000000000009632 (PMC5779762; doi:10.1097/MD.0000000000009632)

**Supplementary Figure 1.** Likelihood mapping of HAV genotype I: first (a), second (b), third (c), fourth (d) and fifth dataset (e). Each dot represents the likelihoods of the three possible unrooted trees for a set of four randomly selected sequences: dots close to the corners represent tree-like phylogenetic signal and those at the sides represent network-like signals. The central area of the likelihood map represents a star-like signal of unresolved phylogenetic information.

**
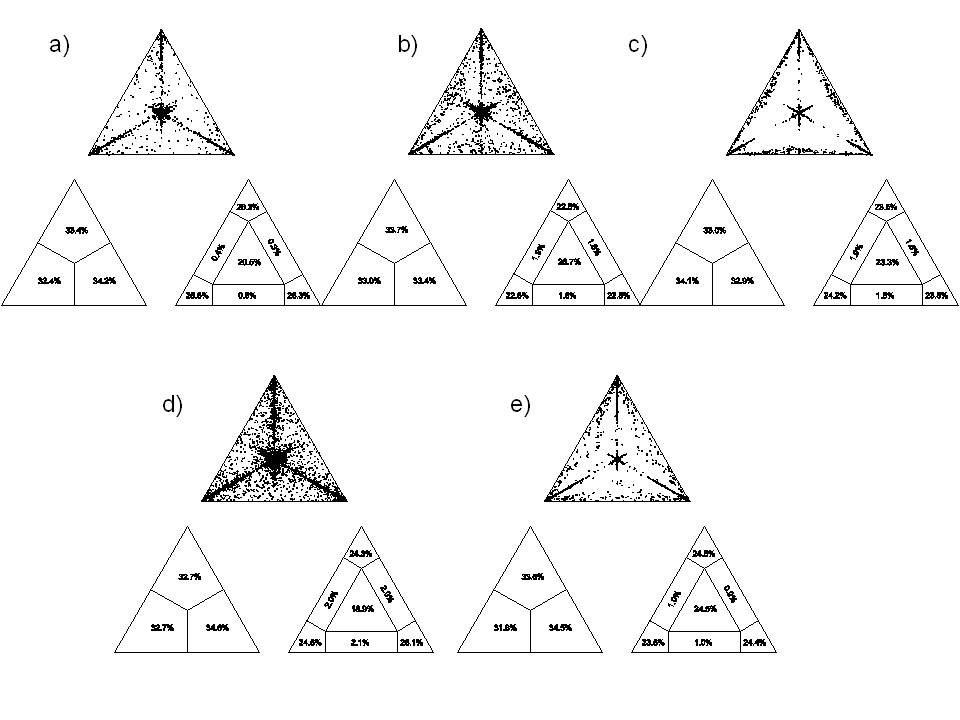
**

**Supplementary Figure 2.** Maximum-Likelihood tree of HAV VP1-2A gene sequences. * along the branches indicating a significant statistical value from bootstrap or sh-LRT analyses; ** indicating a statistical value from bootstrap and sh-LRT analyses. Bulgarian sequences are in bold. The scale bar at the bottom indicates 0.06 nucleotide substitutions per site.


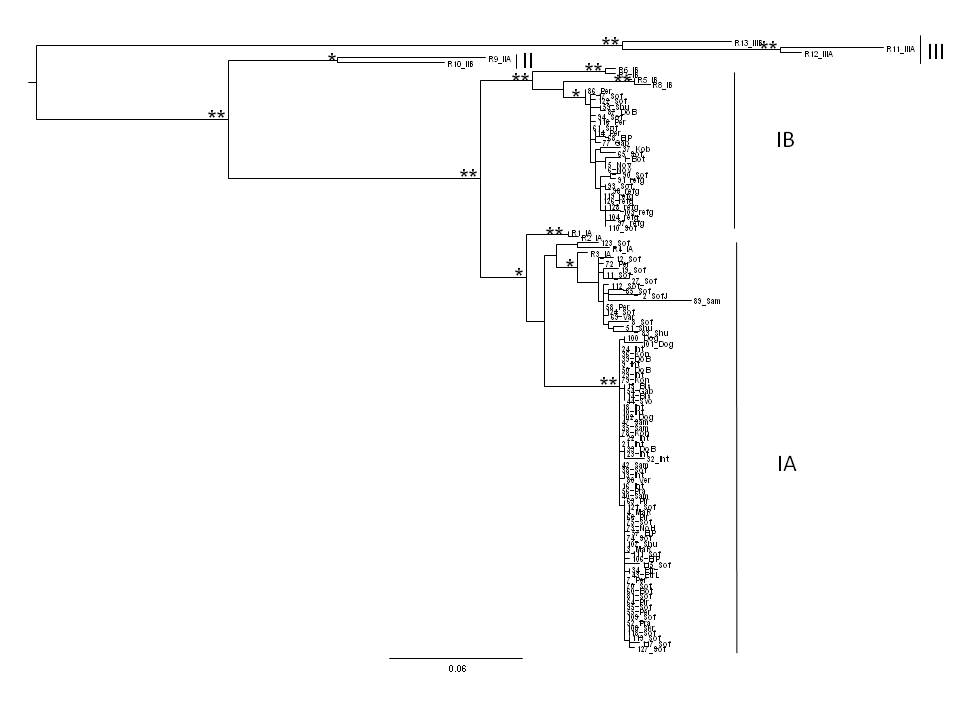

Supplement: Supplemental Digital Content [file medi-97-e9632-s001.doc]
